# A Platform for Mechano(-Electrical) Characterization of Free-Standing Micron-Sized Structures and Interconnects

**DOI:** 10.3390/mi9010039

**Published:** 2018-01-18

**Authors:** Angel Savov, Shivani Joshi, Salman Shafqat, Johan Hoefnagels, Marcus Louwerse, Ronald Stoute, Ronald Dekker

**Affiliations:** 1 Department of Microelectronics, Delft University of Technology, 2628 CD Delft, The Netherlands; shivani.joshi@philips.com (S.J.); ronald.stoute@tno.nl (R.S.); ronald.dekker@philips.com (R.D.); 2 Department of Mechanical Engineering, Eindhoven University of Technology, 5600 MB Eindhoven, The Netherlands; s.shafqat@tue.nl (S.S.); j.p.m.hoefnagels@tue.nl (J.H.); 3 Philips Research, High Tech Campus 4, 5654 AE Eindhoven, The Netherlands; marcus.louwerse@philips.com

**Keywords:** stretchable electronics, stretchable interconnects, MEMS, mechanical characterization, electrical characterization, free standing interconnects

## Abstract

A device for studying the mechanical and electrical behavior of free-standing micro-fabricated metal structures, subjected to a very large deformation, is presented in this paper. The free-standing structures are intended to serve as interconnects in high-density, highly stretchable electronic circuits. For an easy, damage-free handling and mounting of these free-standing structures, the device is designed to be fabricated as a single chip/unit that is separated into two independently movable parts after it is fixed in the tensile test stage. Furthermore, the fabrication method allows for test structures of different geometries to be easily fabricated on the same substrate. The utility of the device has been demonstrated by stretching the free-standing interconnect structures in excess of 1000% while simultaneously measuring their electrical resistance. Important design considerations and encountered processing challenges and their solutions are discussed in this paper.

## 1. Introduction

**Figure 1:**
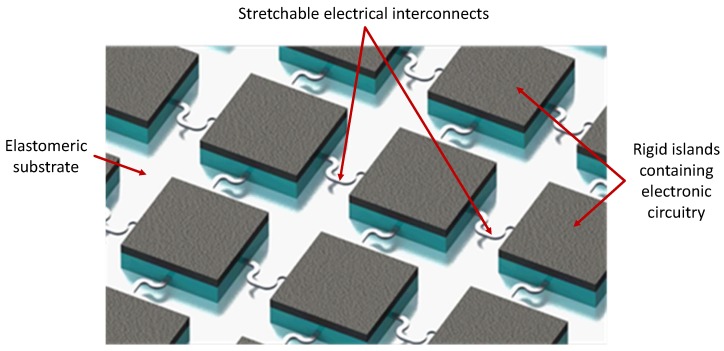
A conceptual illustration of a hybrid stretchable electronics circuit. The rigid islands that contain the electronic functionality are attached to a stretchable substrate and interconnected by (e.g., horse-shoe shaped) free-standing metal interconnects. The overall stretchability of the circuit is mainly determined by the stretchability of the free-standing interconnects.

An increasing number of medical instruments are using micro-fabricated devices as the interface between machines and the human body (or living tissue in general). Examples include smart body patches [1,2], electronic skin [3], implantable devices [4,5], and organ-on-chip devices [6,7]. While biological tissue is soft, bendable and to some extent stretchable, most micro-fabricated devices are hard, rigid, flat and have sharp edges. For the successful integration of living tissue and electronics, the electronic components need to adapt to the mechanical properties of the living tissue. By assembling electronic components on compliant substrates like PDMS (Polydimethylsiloxane) or polyurethane, with mechanical properties close to human tissue, applications such as body patches, neural electrodes [8] and tunable hemispherical cameras have already been demonstrated [9]. These devices are fabricated using, what can be generally characterized as a hybrid approach towards making stretchable electronics: the combination of rigid islands that contain the required functionality attached to a stretchable substrate (Figure 1). A crucial part of such devices are the stretchable interconnects between the rigid islands, since their behavior determines the maximum stretchability and reliability of the entire system.

In most of these hybrid approaches, the planar spring-like interconnects are embedded into the stretchable substrate itself [1,10]. Although this directly isolates the interconnects from each other, it also limits their stretchability. Under large global stretching, high shear stresses build up at the interface between the embedded interconnect and the substrate, resulting in delamination and subsequent failure [11]. For high stretchability applications recent research [12,13,14,15,16] has therefore focused on completely free-standing interconnects that are fully detached from the stretchable substrate, and hence can freely bend and twist out of plane in order to accommodate much larger stretching. In order to design more compliant and reliable stretchable electronic circuits using free-standing interconnects, a better understanding of their mechanical and electrical behaviour under large global stretching is needed. The application area of the structures to be tested is not limited only to stretchable electronic circuits but can include any type of free-standing structures with different application such as for example interconnects used for mechanical isolation of material samples for cryogenic measurements [17].

For the characterization of micro-machined materials, where due to size effects mechanical properties often differ significantly from those at macro scale, test systems have been developed that perform material testing (tensile and bending test) on micron-size samples [18,19,20,21]. Apart from material testing, it is important to study the deformation of the interconnect structure itself, as it would be loaded in the actual application, before fabricating the whole device. For the characterization of such structures, a testing approach is required that allows for the application of very large displacements to test structures with the same size, shape and microstructure as in the final application, while providing a solution to prevent loading of these fragile structures before the test. On-chip testing techniques are well suited for well controlled loading of fragile free-standing structures. However, the currently available on-chip testing techniques [22] do not allow the structures to be tested under very large displacements, requiring a new type of test device and test setup.

In this paper, a test device for electrical and mechanical characterization of free-standing structures suspended over a micron-sized gap is proposed. The fabrication flow of the device allows a variety of free-standing structures with different geometries to be fabricated and tested, making it a versatile testing platform. The device consist of two beams between which the structures are suspended. When the beams are moved apart, the structures between them are loaded and the resulting deformations can be observed by means of optical microscopy, scanning electron microscopy or profilometry. For a complete characterization, the electrical resistance is measured in situ as a function of displacement. The device is fabricated using standard silicon micromachining which allows for high precision and reproducibility of the fabrication process, while using already available manufacturing equipment. Such processes and equipment are typically used to process micron-size stretchable interconnects and structures. Large number of stretchable electronic circuits are built by using fabrication technologies different from silicon micromachining using polymer or textile substrates [23,24]. It is important to point out that only materials that are compatible with the silicon micromachining fabrication flow can be used. This means structures built from materials such as metals that are sputtered or evaporated can be easily studied, while structures made from organic materials do not lend themselves to testing with the proposed device since they are not compatible with the fabrication flow.

## 2. Concept Discussion

### 2.1. Requirements

The characterization of the mechanical properties of micron-size interconnects subjected to large global displacement, demands for the fulfillment of a combination of requirements for the test setup. Upon review of the available MEMS-based actuators [22], no suitable technology that can provide both the required stretchability and displacement precision is reported. This eliminates the possibility of simultaneously manufacturing the test structures and actuators on the same chip, as commonly done for testing micron-scale structures. Since the size of the interconnects is very small, their direct handling is impossible without causing damage. Therefore, the test device onto which the interconnects are to be fabricated should be designed in a way that allows for safe transport and mounting in the test setup. Furthermore, a possibility to simultaneously measure the resistance of the individual interconnects needs to be integrated in the test device. Additionally, the device has to be designed such that various deformations modes of different types of structures can be explored. In order to do so, different structure geometries spanning different test gaps (free-standing lengths) need to be fabricated. Finally, the fabrication process chosen for the manufacturing of the test devices should not affect the microstructure and the resulting mechanical properties of the interconnects.

### 2.2. Design

**Figure 2:**
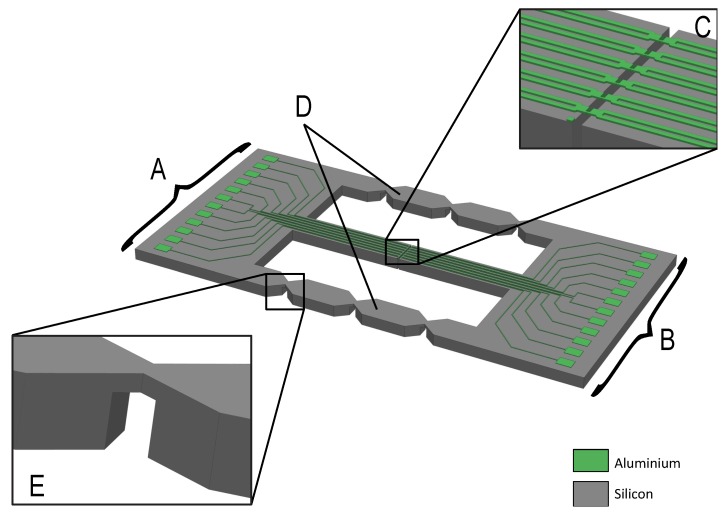
Schematic representation of the test chip. Here, A and B represent the main islands in between which the interconnects are suspended, shown in C. These interconnects are kept in place by the two temporary support beams, represented by D, each containing three v-notches. In order to break these notches easily the thickness of the Si is reduced, as shown in E.

Keeping the above requirements in mind, a test device for testing of the micron-size interconnect structures is designed (Figure 2). The device consists of two main islands (A and B in Figure 2) between which the free standing interconnects are suspended (C in Figure 2). To perform the tensile test, the islands are fixed in a micro tensile stage, in such a way that the islands can move independently in a controlled and precise manner. On the islands, metal tracks that are connected to the interconnects are designed in such a way that electrical measurements can be carried out during the tensile test. The miniaturized design allows for 4-probe electrical resistance of all six parallel interconnects, simultaneously with applied stretch, thus conveniently allowing to study the evolution of electrical resistance during quasi-static and cyclic loading.

In order to execute the test, the two islands (A and B) need to be moved independently. However, the interconnects suspended between them are very fragile, and therefore any uncontrolled movement before the test should be avoided. To ensure safe transport and straightforward mounting into the test stage, the chip is designed as a single rigid frame where the islands A and B are connected by the temporary support beams D (Figure 2) that prevent spurious motion between the main islands. After the device has been safely mounted into the test stage, the support beams D are broken off, and the chip is split into two parts that move independently. To avoid the risk of spurious motion during fracture due to a large release of energy, notches E (Figure 2) are designed in the support beams. Since the beams are weaker at these notch locations, they will break at those locations instead of at any arbitrary point in the structure of the test device. The breaking point locations have been selected in such a way as not to damage the main islands or the free standing interconnects.

## 3. Experiment

The manufacturing technology that was used for the fabrication of the test devices is based on the Flex-to-Rigid (F2R) technology [25]. The F2R platform is used in the manufacturing of smart minimally invasive surgical instruments such as intravascular ultrasound (IVUS) catheters [26], where arrays of MEMS ultra-sound transducers and driver electronics need to be packed in the very small volume available at the tip of the instrument. To allow for the manufacturing of the free-standing interconnects, the fabrication flow for the test chips has been modified compared to the fabrication flow for the F2R technology [25,26].

**Figure 3:**
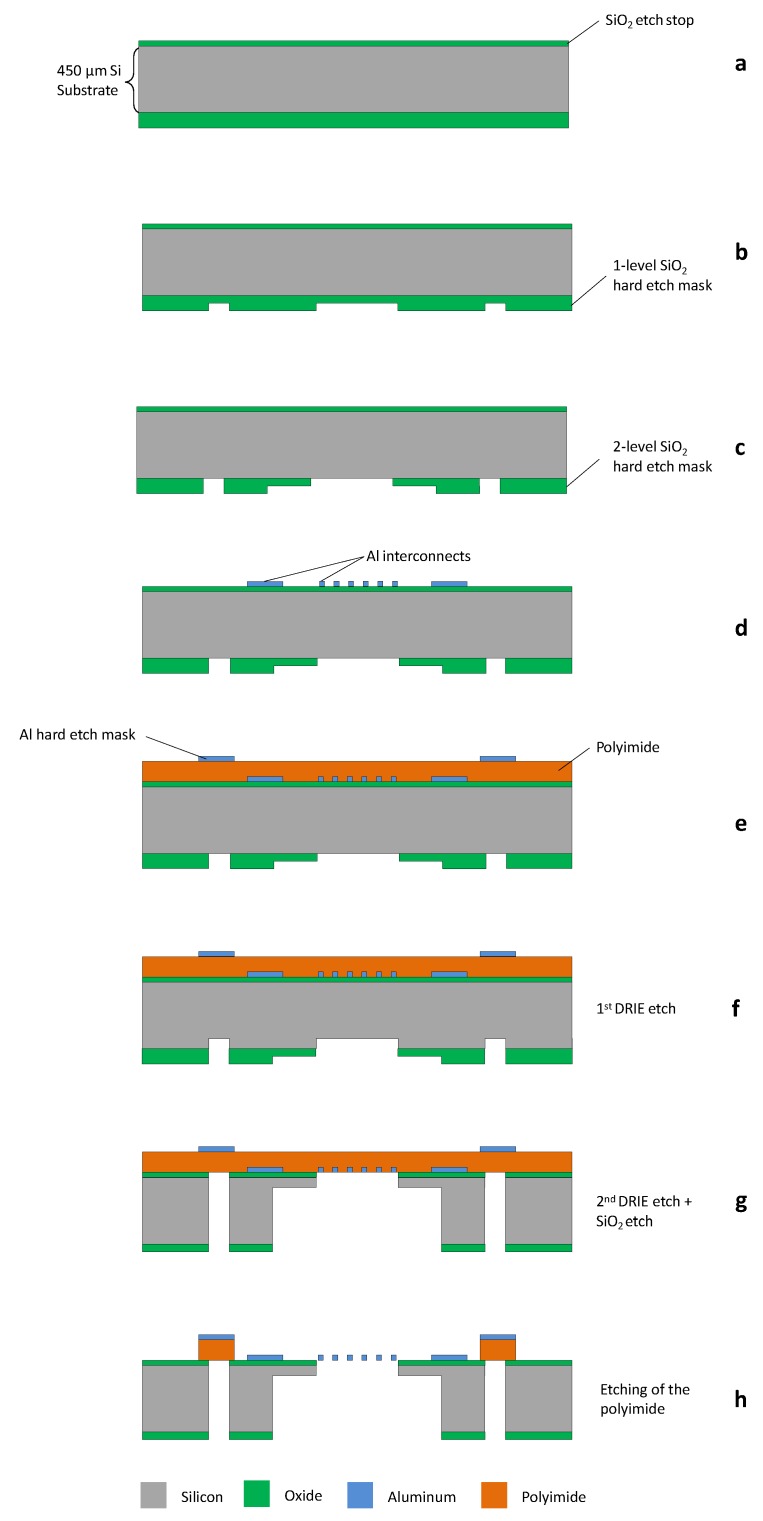
Cross-sectional view of the fabrication flow of the test device.

The fabrication begins with the deposition of a 1 μm and 5 μm thick layer of PECVD silicon oxide (Novellus PECVD concept one) on the front and back side of a 150 mm double side polished 400 μm thick Si substrate (P-doped, Boron <100>), respectively (Figure 3a). The backside SiO${}_{2}$ is patterned (3.5 μm HPR504 positive resist, OCG Microelectronic Materials N.V, Energy 160 mJ/cm${}^{2}$, ASML PAS5500 stepper) into a two level hard etch mask (Figure 3b,c) and etched (STS ACP tool, CF${}_{4}$ plasma, 9 min). This two level hard etch mask is used towards the end of the fabrication flow to define the outer shape, the gaps underneath the freestanding interconnects, and the thinned down areas of the device. After etching of the back side oxide, the resist mask is removed in acetone. The front side processing starts with sputter deposition of 200 nm thick layer of aluminum (Veeco 2 Nexus, UHV system with 99.99999% Al target purity and 2 nm/s deposition rate), patterned (1.2 μm SPR660 positive resist, Energy 160 mJ, ASML PAS5500 stepper) and developed (AZ400K developer). Using this resist mask, the aluminum is dry etched (STS ICP Clustertool, Cl${}_{2}$ chemistry plasma, 5 mTorr) defining the desired interconnect patterns along with the re-routing layer and the bond pads (Figure 3d). The resist mask is removed by dry etching in a barrel by O${}_{2}$ plasma (600 W, 150 ${}^{\circ }$C, 15 min, IPC9200 from PVA Tepla). Next, a 5.2 μm thick layer of polyimide (PI 2611, HD Microsystems, Neu-Isenburg, Germany) is spin coated at 3000 rpm for 45 s and cured at 275 ${}^{\circ }$C for 3 h in a nitrogen atmosphere (KOYO Thermo Systems Co.Ltd., Nara, Japan). After curing of the polyimide a 200 nm thick layer of aluminum is sputter coated (Veeco 2 Nexus) on top of the polyimide. The Al layer is patterned (3.6 μm HPR504 positive resist, Energy- 160 mJ, ASML PAS5500 stepper) and wet etched at 30 ${}^{\circ }$C in a commercially available wet etchant (PES 77-19-04, BASF, Ludwigshafen, Germany) consisting of phosphoric acid (H${}_{3}$PO${}_{4}$), nitric acid (HNO${}_{3}$) and acetic acid (CH${}_{3}$COOH), with an etch rate of 95 nm/min (Figure 3e). This Al layer is used as a hard etch mask at the end of the fabrication process to etch the underlying polyimide layer. In the next step, the wafer is etched from the back side by means of Deep Reactive Ion Etching (DRIE), using the silicon oxide hard etch mask defined earlier. In order to form narrow and deep trenches, the Si etching is performed in two steps.

First, an advance etch of 200 μm is performed (SF${}_{6}$ + C${}_{4}$F${}_{8}$ gases, 20 min in STS PEG Tool) using the first level of the hard etch mask (Figure 3f). This is followed by a short silicon oxide etch (CF${}_{4}$ chemistry plasma, 9 min in STS APS Tool) which opens up the second level of the hard etch mask that defines the wider trenches. Using this second level mask, the silicon etch is continued (SF${}_{6}$ + C${}_{4}$F${}_{8}$ gases, 20 min DRIE in STS PEG Tool) until the oxide on the front side of the wafer is reached and subsequently etched (CF${}_{4}$ gas, 22 min in STS APS Tool). The trenches formed by the DRIE define the general shape (outline) of the silicon islands. This two-step approach serves two purposes. Firstly, it allows the relatively narrow trenches to be defined on the back side of the wafer, and be precisely transferred through etching to the front side. Secondly, it also allows the substrate to be thinned down at selected locations. This is especially useful in cases where the breaking points of the support beams are to be defined (notches E in Figure 2). After the second and final DRIE step (Figure 3g) the test devices are completely shaped.

The polyimide (PI) layer on the front side of the wafer separates the test devices from the bulk of the wafer, and holds them into place. In the final processing step, the PI is dry etched in an oxygen plasma (6 min RIE in STS ICP tool), using the pre-patterned aluminum hard etch mask (Figure 3h). After etching the devices remain attached to the frame of the wafer by a small number of PI tabs. By laser cutting the PI tabs, these test devices can be removed from the main frame for mounting in the test stage. The rigid temporary support beams prevent loading of the interconnects, thereby reducing the chance of damaging the delicate structures.

## 4. Results and Discussions

### 4.1. Fabrication of Test Device

**Figure 4:**
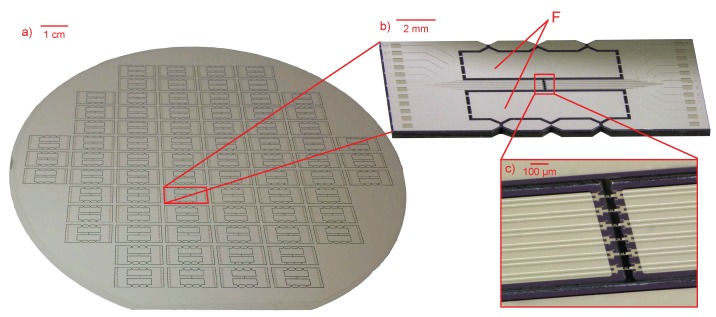
(**a**) A photograph of a completely processed wafer containing a number of test chips, held in the wafer frame by means of PI tabs; (**b**) A single test chip taken out of the wafer frame. The silicon areas next to the beams, denoted by F, are sacrificial and are removed before mounting; (**c**) A magnified image of the test structures that are freely suspended between the beams of the test chip.

To test the manufacturing feasibility of the proposed characterization platform, a number of highly-stretchable free-standing test structures with different geometries were fabricated. Other types of free-standing structures can be manufactured and tested as well. The design of the test chips allowed for 76 devices to be fabricated onto a single 150 mm diameter Si wafer (Figure 4a). At the end of the fabrication process, the test devices remain suspended to the main frame of the wafer, held in place only by means of the polyimide tabs. These PI tabs allows for easy separation and removal of the devices from the wafer without incurring damage to the test structures unlike in a dicing process.

**Figure 5:**
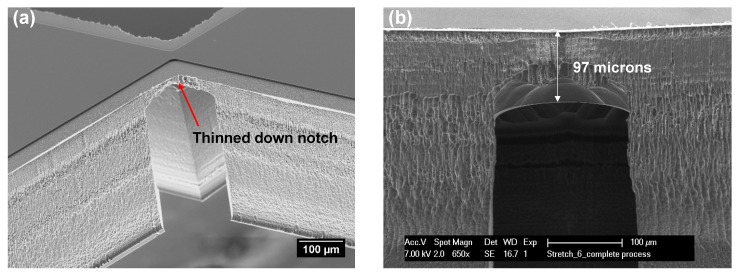
(**a**) SEM image of the notching points of the beams; (**b**) The pre-designed reduction of thickness of the temporary support beams at the v-notch points is clearly visible in the SEM cross section.

**Figure 6:**
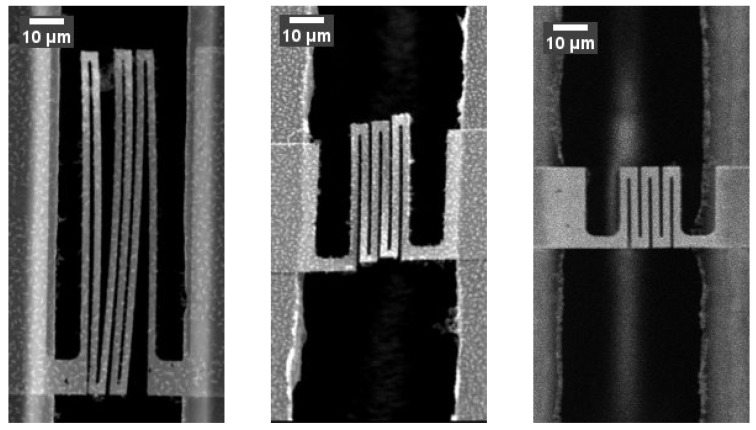
SEM pictures showing free standing test structures with different dimensions spanning a 50 μm test gap fabricated on the same substrate. The picture demonstrates that different test structures can be fabricated on the same substrate using the same fabrication process.

The two step DRIE process allows for the formation of thinned down areas. This has been utilized to reduce the thickness of the supporting beams at the v-notch points (Figure 5). This allows for a controlled breaking of the silicon beams without the need to use excessive force. The two step DRIE also allows for test chips with different width of the test gaps to be manufactured on the same wafer. Since both the test structure geometry and test gap widths are defined by lithography, test devices with different structures were fabricated on the same wafer (Figure 6).

Various processing issues were encountered during the fabrication of the test chip. A short discussion of the most relevant issues and their solutions are described in the following section.

### 4.2. Fabrication Challenges

**Figure 7:**
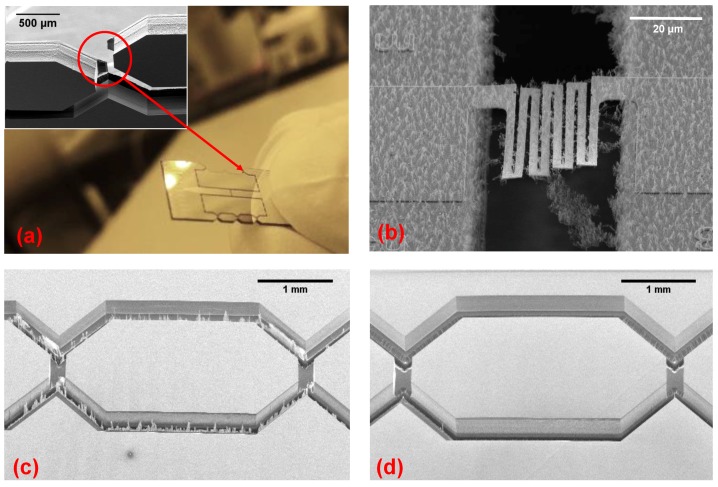
(**a**) A test device with a premature (directly after processing) breaking of the supporting beams at the v-notch points (see Figure 5) due to excessive thinning (fracture of the v-notch shown in inset); (**b**) Free standing interconnects after PI etch covered with residues; (**c**) SEM micrograph showing the back side of a test chip where remnants in the forms of spikes are visible, which are caused by the passivation that has been left over from the first step of the DRIE etch; (**d**) SEM micrograph of the same area of the device after an isotropic silicon etch shows the successful removal of the remnants.

One of the most important issues is uncontrolled thinning of the supporting beams at the v-notch points of the device, as this affects the overall mechanical stability of the test device. If the notches are too thin, the chip will disintegrate when handled or even during processing (Figure 7a). Conversely, if the beams are too thick, too much force will be needed to break them, resulting in uncontrolled separation or elastic recoil, which may pre-deform the interconnects. Therefore, the first DRIE etch was carefully optimized in such a way that the second DRIE step stops precisely at the correct thickness of the notches. It was determined that for this design and geometry thickness of 100 μm resulted in v-notches that were easily breakable, while still being sturdy enough to sustain the handling of the device (Figure 5).

The final step in the releasing of the interconnects includes the dry etching of PI in an oxygen plasma. After the etching of the PI is completed, remnants are observed on the surface and around the interconnects (Figure 7b). Remnants after etching of PI have been observed before [27,28], and they have been traced back to PI types that include primers containing siloxane groups which cannot be etched in an oxygen plasma. In this work a polyimide (PI2611, HD Microsystems, Neu-Isenburg, Germany) without primer was used, ruling out this possibility. A possible explanation for the residues was the sputter deposition method of the Al metal mask, due to which some metal particles get implanted in the PI. These metal particles are not etched in the O${}_{2}$ plasma during PI etch and form residues. Due to the small size, non-dense structure and the density of the observed remnants it is reasonable to assume that they do not have a significant effect on the working mechanism of the interconnects. However, in order to avoid the formation of the remnants in the future, it was proposed to dry etch the Al mask before PI etching. Dry etching of Al mask with a slight overetch resulted in a residue-free PI etching. The slight overetch erodes the surface of the PI where the Al mask remnants are embedded [29].

Another issue observed during processing are “spikes” in the trenches on the back side of the wafer left after the two step DRIE (Figure 7c). Due to incomplete removal of the passivation deposited during the first step of the DRIE (etching of narrow trenches), there are areas where the silicon is not completely etched after the second DRIE, resulting in the formation of these “spikes”. These residues generally do not present a problem to the functioning of the chip, but can pose a health hazard if inhaled and serve as a source of contamination for the processing equipment. The problem has been resolved by executing a short isotropic silicon etch after the anisotropic DRIE etching has been completed (Figure 7d).

### 4.3. Test Setup and Mounting

Since mounting and testing of highly stretchable free-standing structures is not trivial and the design of the test chip is aimed at addressing the handling challenges, to demonstrate the capabilities of the platform the test devices were mounted and characterized. The following description of the mounting and measurement of the test structures is meant to serve merely as a demonstration of the capabilities of the test platform and is not intended as a discussion of the properties of the specific structure. For a more detailed discussion of the design and electro-mechanical properties of the structures shown, please refer to [15].

**Figure 8:**
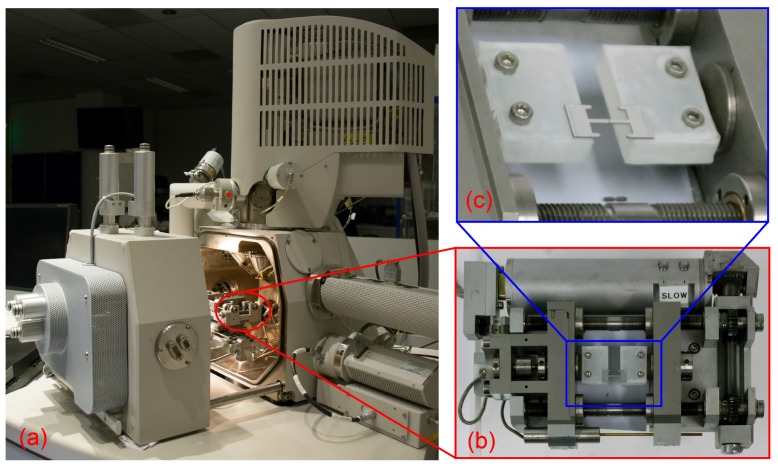
(**a**) An SEM with a commercially available micro-tensile stage mounted in it. (**b**) A top view of the micro-tensile test stage with the test chip mounted on it. (**c**) A perspective view of the mounted test chip. The temporary support beams that provide mechanical stability during transport and mounting of the test chip have been removed.

**Figure 9:**
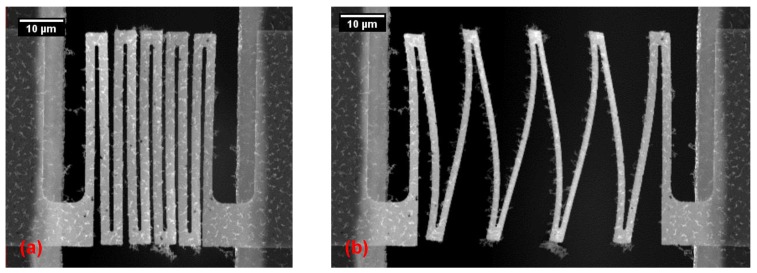
Two in situ SEM images showing the same interconnect in (**a**) initial and (**b**) stretched condition. The images demonstrate that the test structures fabricated in the test device can be strained inside the SEM as designed.

The characterization tests are performed by first singulating test devices from the wafer by cutting the polyimide tabs. Next, this device is clamped to two acrylic blocks providing a flat top surface (Figure 8). The device is attached to the blocks by gluing both ends on the top surface of the acrylic clamps with a UV-curable adhesive (Loxeal 58-11) (Figure 8b). The use of UV-curable adhesive allows to ensure a good alignment with respect to the loading direction. Once a precise alignment of the device with respect to the clamps is achieved, the adhesive is UV cured. Subsequently, the silicon beams (Figure 2E) that keep the two islands of the test-chip connected with each other, are broken to allow for free movement of the Si islands, so that the interconnects can be stretched. The crystallographic orientation of the silicon substrate (<100>) is aligned to the width of the notches to allow crack propagation along it to break the beams in a controlled fashion (Figure 8c). The notched silicon beams-based test design ensures that the test device is robust with respect to in-plane loads, while a controlled small amount of downwards force applied in the center of the beams generates sufficient moment in the notches to break them. Furthermore, since the beams connect to the test interconnects through the main islands (Figure 2A,B), which are clamped in the test setup, the transmission of the energy released during the beam breaking to the test interconnect is minimized. After breaking the beams, the two test device islands are now only connected by the freestanding interconnects and are free to be displaced with respect to each other to perform the tensile test (Figure 9). The tensile stage used in the current experiment allows the application of uniaxial stretching. In most of the applications of stretchable electronics multi-axial global strain is usually encountered. The limitation in this respect is posed by the tensile stage itself and is not an inherent limitation of the test chip. By designing a different tensile stage setup multi-axial strain can be applied to the test structures without changing the design of the test chips.

**Figure 10:**
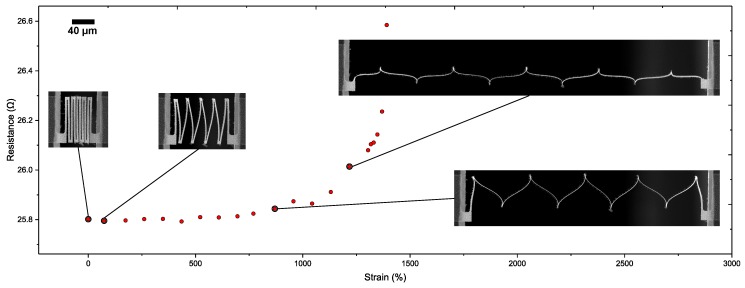
To demonstrate the capabilities of the test platform a measurement of the a graph of the resistance value of a single test structure/micro interconnect as a function of applied displacement/strain has been presented. The test was performed under an optical microscope to avoid any effects of the SEM electron beam with the electrical resistance measurements. To clearly show the deformation states of the structures SEM micrographs have been included.

To illustrate what type of measurements can be performed with the proposed test chip platform, a high resolution in situ mechanical test was performed inside a large chamber SEM (Figure 8) to determine the maximum reversible and irreversible stretch of the free-standing interconnects. After each loading step (of increasingly higher stretch), the interconnects are unloaded to the initial configuration to study the effects of plastic deformation. The results show that with stretchability of up to 623% the interconnects can recover their original shape (Figure 10), thus indicating that the stretch is reversible. Beyond this point the effect of plasticity can be clearly seen in the unloaded interconnect shape (Figure 10). The interconnects are further stretched almost to a straight wire (Figure 10), at a stretchability of 1240%, beyond which point the interconnects fracture. Furthermore, four-probe electrical resistance measurements were preformed with simultaneous mechanical loading to study the effects of deformation on the evolution of electrical resistance. The test was performed under an optical microscope to avoid any interference of the SEM electron beam with the electrical resistance measurements. The results show that the electrical resistance stays stable even into the plastic regime, e.g., at stretchability of 1108% the electrical resistance varies by only by 0.4% with respect to the initial configuration. Only once the interconnect is completely stretched out in extreme plasticity, the electrical resistance varies by 3%. Two regimes in the electrical resistance evolution (Figure 10) can be identified, as also discussed by [30,31,32]. In the first regime till 623%, in which the interconnect stretches elastically, the electrical resistance increase is minute. Beyond 623%, due to the onset of plasticity and possible damage accumulation (not visible in SEM images), the electrical resistance starts to increase exponentially till the interconnect fracture. For more detailed discussion on the testing of similar free-standing structures, please refer to [15]. These successful mechanical and electrical characterization tests demonstrate the utility of the test chip platform discussed in this paper.

## 5. Conclusions

The development of stretchable devices and circuits (by means of hybrid technology) requires accurate characterization of the mechanical and electrical properties of all the components in these systems. This is especially true for structures such as the stretchable interconnects as they form a crucial part of the system. Due to further developments in the field of stretchable electronics, there is a push towards high-density stretchable electronics devices, which requires small (micron-sized) interconnect footprints and high stretchability. The testing of such micron-sized free-standing interconnects is known to be challenging. In this paper, we present a micro-fabricated test device that allows highly stretchable/compliant micron-sized structures to be handled so that they can be successfully mounted and tested in a micro-tensile stage. The test device in which the structures to be tested are suspended is robust and rigid. The device is designed in such a way that after mounting it on the test set-up, it is split into two independently moving parts by the controlled removal of two supporting silicon beams. The concept was successfully demonstrated by repeatedly stretching micron-size interconnect to 2000% reversibly, while the resistance is measured simultaneously. Finally, the design of the device allows for the fabrication of free-standing test structures with different shapes, and spanning different test gaps in the same wafer. The test chip platform is generic, as it allows for the integration of various kinds of microfabricated structures such as strain gages, displacement sensors and actuators, and even active electronics.
